# Mélanome cutané épais: facteurs de mortalité et de survenue de métastases

**DOI:** 10.11604/pamj.2014.18.44.2812

**Published:** 2014-05-12

**Authors:** Mariam Tarwate, Hakima Benchikhi, Latifa Adarmouch, Abdelatif Benider, Mohamed Amine, Soumya Zamiati, El Hassan Boukind

**Affiliations:** 1 Service de dermatologie vénérologie CHU Ibn Rochd Casablanca; 2 Département de médecine communautaire CHU Mohamed VI Marrakech; 3 Service d'oncologie CHU Ibn Rochd Casablanca; 4 Service d'anatomopathologie CHU Ibn Rochd Casablanca; 5 Service de chirurgie plastique et réparatrice CHU Ibn Rochd Casablanca

**Keywords:** Mélanome, épais, métastases, mortalité, Breslow, Melanoma, thick, metastases, mortality, Breslow

## Abstract

Au Maroc peu d’études ont été menées concernant les facteurs pronostiques du mélanome. L'objectif de cette étude était d'identifier les facteurs de survenue de métastases viscérales à distance à 1an et de mortalité à 2ans chez des patients atteints de mélanome cutané épais. Ont été colligés rétrospectivement tous les cas de mélanomes suivis au service de dermatologie et d'oncologie du CHU de Casablanca de 2006 à 2010. On été inclus les patients qui présentaient un mélanome à localisation cutanée et/ou muqueuse buccogénitale, confirmés histologiquement ayant un Breslow>2mm. Ont été exclus les patients présentant des métastases viscérales à distance au moment du diagnostic. Les données socio-démographiques, cliniques et thérapeutiques ont été recueillies au niveau des dossiers; celles relatives à l’évolution ont été complétées par contact téléphonique. L'analyse statistique était de type uni et bivarié. Soixante patients ont été inclus:41hommes et19femmes (sexe ratio de 2,15). La moyenne d’âge était de 60 ans (17-96). L'indice de Breslow était en moyenne de10mm+/-7et78%des patients(32) avaient un Breslow>4mm. Vingt trois patients (38,3%) avaient des adénopathies régionales. Quatorze patients ont présenté des métastases à distance après 1an (23,3%) et13patients étaient décédés après 2ans d’évolution (21,7%). Les facteurs associés à la survenue de métastases à distance étaient l'indice de Breslow. Des métastases à 1an ont été retrouvées chez 61,5%des patients avec un Breslow >10mm contre18,8% avec un Breslow

## Introduction

Le mélanome cutané est une tumeur redoutable, il ne représente que 5% des cancers cutanés mais entraîne 75% des décès qui leur sont liés [1]. Son incidence est en augmentation dans les pays à race blanche (3 à 7% chez les causasiens) [2], avec une augmentation simultanée de la mortalité [3]. Dans les populations hispaniques et afroaméricaines, l'incidence est plus faible mais ces groupes ethniques sont plus à risque de développer des mélanomes à potentiel métastatique plus élevé avec un plus mauvais pronostic [4, 5]. En Afrique et au Maghreb, les mélanomes sont peu fréquents, mais souvent épais de type nodulaire ou acrolentigineux avec des métastases ganglionnaires précoces [6–10].

Les facteurs pronostiques en général sont bien connus. Ils sont représentés essentiellement par l’épaisseur tumorale et l'ulcération qui sont les facteurs prédictifs majeurs de survie à 5 ans. Les autres facteurs sont l'index mitotique, le nombre de ganglions métastatiques et la présence de métastases viscérales par rapport aux métastases non viscérales qui sont de meilleur pronostic [11].

Peu d’études se sont penchées sur l’étude des facteurs pronostiques des mélanomes épais dans le contexte maghrébin. D'où le but de notre étude qui était d'identifier les facteurs de survenue de métastases à distance à 1 an, et de mortalité à 2 ans dans le mélanome cutané épais.

## Méthodes

Il s'agit d'une étude rétrospective menée aux services de dermatologie et d'oncologie du CHU Ibn Rochd de Casablanca colligeant les cas de mélanome suivis entre janvier 2006 et décembre 2010. Ont été inclus les patients qui avaient un mélanome à localisation cutanée et/ou de la muqueuse buccogénitale, confirmé histologiquement, et ayant un indice de Breslow ≥2mm. Les patients qui avaient des métastases viscérales ou ganglionnaires à distance au moment du diagnostic ont été exclus de l’étude.

Les données recueillies sur les dossiers médicaux étaient les suivantes: l’âge, le sexe, le délai entre l'apparition de la lésion et le diagnostic, l'aspect clinique, la taille, la localisation, l'aspect histologique (type anatomoclinique, l'indice de Breslow, l'ulcération histologique, mitoses), le traitement reçu, l'apparition de métastases ganglionnaires régionales, et de métastases à distance et le délai de leur apparition, le décès et le délai de sa survenue. Les patients ou leurs familles ont été contactés par téléphone pour avoir l'information sur le décès lorsqu'elle ne figurait pas dans le dossier.

Les événements étudiés étaient le décès à 2ans et la survenue de métastases à distance à 1an. L'analyse statistique était réalisée à l'aide du logiciel SPSS version 16.0 avec analyse de type uni et bivarié. Le seuil de signification était fixé à 0,05.

## Résultats

Soixante six cas de mélanomes ont été recensés durant ces 5 ans. Six patients ont été exclus car ils présentaient déjà des métastases à distance au moment du diagnostic: 4 patients avaient des métastases hépatiques, un avait des métastases pulmonaires, cérébrales et osseuses, et un des métastases cutanées à distance. Soixante cas ont rempli tous les critères, tous avaient un Breslow 2mm. Les caractéristiques sociodémographiques et cliniques figurent dans le Tableau 1. Les localisations concernées étaient: la plante dans 41,6%(25cas), les orteils dans 10%(6), les MI 16,66%(10), le visage et l'ongle dans 8,33%(5 cas) chacun. L'aspect de tumeur bourgeonnante ou ulcérobourgeonnante était retrouvé chez 63,3% (38 cas), un aspect nodulaire chez 11 cas, une ulcération chez 4 cas, une nappe pigmentée chez 4 cas et une atteinte unguéale dans 5 cas à type de mélanonychie avec onychodystrophie. Le type anatomoclinique nodulaire était retrouvé chez 68,3%(41), l'acrolentigineux chez 13,3%(8), le mélanome à extension superficielle et le mélanome unguéal représentaient 8,3% (5 cas) chacun. Dans un cas le type anatomoclinique n’était pas précisé. L'indice de Breslow était en moyenne de 10mm +/-7 et 78% des patients (32 cas) avaient un Breslow >4mm (Tableau 1).

**Tableau 1:** Caractéristiques cliniques et histologiques des patients

	Moyenne+ /- Ecart type	%
Age	60 +/- 1,62	
**Sexe**		
Hommes	41	68
Femmes	19	32
Délai diagnostique	17,7 +/-20,4	
**Mélanome sur naevus**		
Oui	15	45,4
Non	18	54,5
**Aspect clinique**		
Tumeur B/UB	38	63
Nodule	11	18
Nappe pigmentée	7	11,6
Autre:	4	6,6
Taille tumorale (mm)	42 +/- 26,4	
Indice de Breslow (mm)	10,5 +/- 7	
≤4	9	22
>4	32	78
**Ulcération histologique**		
Oui	26	65
Non	14	35
**Type anatomoclinique**		
Nodulaire	41	69,5
Acrolentigineux	8	13,5
SSM	5	8,5
Unguéal	5	8,5

Vingt huit patients avaient développé des métastases ganglionnaires régionales dont 13 (46%) au cours de la première année de suivi. Chez 14 patients cette information n’était pas précisée. Vint-neuf patients ont développé des métastases à distances dont 14 après 1 an (46%). Vingt-trois patients étaient décédés dont 13 après 2ans d’évolution (56%).

Les facteurs associés à la survenue de métastases à distance étaient l'indice de Breslow: des métastases à distance à 1an ont été retrouvées chez 61,5%des patients ayant un Breslow >10mm contre 18,8% chez ceux ayant un Breslow

Les facteurs associés à la mortalité à 2 ans étaient l'existence de métastases ganglionnaires régionales: 56,2% des patients qui les avaient étaient décédés contre 16% chez ceux qui n'en avaient pas (p=0,007) et la survenue de métastases à distance (12% des patients qui ont développés des métastases à distance étaient décédés et aucun décès n'a été retrouvé chez les patients qui n'avaient pas de métastases à distance (p=0,003) (Tableau 2).

**Tableau 2:** Facteurs associés à la survenue de métastases à 1 an et au décès à 2 ans

	Métastases à 1 an			Décès à 2ans		
	N	%	p	N	%	p
**Age**						
≤ 60 ans	8	36,4	0,943	7	31,8	0,987
> 60 ans	6	37,5		6	31,6	
**Sexe**						
H	11	42,3	0,472	11	40,7	0,156
F	3	25		2	14,3	
**Délai diagnostic**						
≤ 1an	6	27,3	0,166	9	34,6	1
>1an	7	50		4	28,6	
**Breslow**						
≤ 10mm	3	18,8	0,027	4	22,2	0,418
> 10mm	8	61,5		5	41,7	
**Type anatomoclinique**						
Nodulaire	11	45,8	0,288	8	32	1
Autre que nodulaire	3	23,1		5	33,3	
**Ulcération histologique**						
Oui	7	50	0,391	5	35,7	1
Non	4	33,3		4	36,4	
**Métastases ganglionnaires régionales**						
Oui	8	50	0,152	9	56,2	0,007
Non	6	27,3		4	16	
**Curage ganglionnaire**						
Oui	6	40	0,681	6	40	0,485
Non	7	33,3		7	29,2	
**Métastases à distance**						
Oui				12	48	0,003
Non				0	0	

## Discussion

Dans cette étude nous avons retrouvé que, dans les mélanomes épais, l'indice de Breslow était corrélé à la survenue de métastases à distance à 1 an et que les facteurs associés significativement à la mortalité à 2 ans étaient la présence de métastases ganglionnaires régionales et de métastases à distance. L'indice de Breslow constitue donc le facteur pronostic majeur dans cette étude comme dans la littérature.

Un mélanome a été considéré dans notre étude comme épais si le Breslow dépassait 2mm l'indice de Breslow était corrélé à la survenue de métastases à distance à 1 an et que les facteurs associés significativement à la mortalité à 2 ans étaient la présence de métastases ganglionnaires régionales et de métastases à distance. L'indice de Breslow constitue donc le facteur pronostic majeur dans cette étude comme dans la littérature.

Un mélanome a été considéré dans notre étude comme épais si le Breslow dépassait 2mm, sachant que dans le contexte hospitalier nous somme plus confrontés aux mélanomes épais qu'aux mélanomes fins.

Une étude allemande sur 63 cas de mélanomes a retrouvé que la positivité du ganglion sentinelle était le facteur majeur de survie dans les mélanomes épais >4mm 12 sachant que dans le contexte hospitalier nous somme plus confrontés aux mélanomes épais qu'aux mélanomes fins.

Une étude allemande sur 63 cas de mélanomes a retrouvé que la positivité du ganglion sentinelle était le facteur majeur de survie dans les mélanomes épais >4mm [12], ceci est conforté par une étude japonaise sur 291 mélanomes épais ou l'augmentation de l'indice de Breslow et la positivité du ganglion sentinelle sont associés à une mauvaise survie globale [13]. Pour d'autres c'est la nécrose tumorale qui est un élément de mauvais pronostic dans les mélanomes >4mm.

Cette épaisseur tumorale très importante avec un indice de Breslow moyen de 10mm est un élément alarmant, surtout que 78% des patients avaient un Breslow dépassant les 4mm. Dans une cohorte aux USA ayant étudié tous les mélanomes cutanés entre 1988 et 2006, 153124 mélanomes ont été recensés. L'indice de Breslow était ≤1 mm chez 70% et >4mm chez 5% seulement [14]. Dans notre série, le retard diagnostique peut contribuer en grande partie à cette épaisseur, quoi que le délai moyen de diagnostic était de17,7 mois +/-20,4. Les patients consultent souvent tard à cause du caractère indolent des lésions, surtout si elles sont localisées dans des endroits cachés (localisation plantaire et des orteils comme dans 52% des cas de notre série), à cause de l'ignorance et de l'analphabétisme, le bas niveau socioéconomique(NSE)et la difficulté d'accès aux structures de soins.

En comparant avec des études Sudafricaine et Tunisienne, dans lesquelles le délai diagnostique est similaire ou supérieur (18,5% et 36% respectivement) on retrouve un Breslow moins épais (3,17 et 8,34 mm) (Tableau 3). Ceci suggère l'existence d'autres facteurs de risque de mélanome épais. La prédominance du type nodulaire qui représente 69,5% des cas dans notre série serait très probablement incriminée dans cette épaisseur tumorale. Ce type anatomoclinique est considéré actuellement par certains auteurs comme une entité agressive à part et non seulement une phase de croissance verticale; dans une étude récente le MN représentait 14% des mélanomes invasifs, mais était responsable de 43% des décès pour un total de 57.461 personnes-années de suivi, par comparaison avec le mélanome à extension superficielle qui représentait 56% des mélanomes invasifs mais responsable de 30% seulement des décès[15].

**Table 3:** Comparaison d’études africaines et maghrébines

Etude	Nombre	Délai diagnostique (mois)	Breslow (mm)	Type anatomoclinique
**Swan**				42% MAL
Afrique du Sud (2003)	40	18,5	3,17	29% MN
				25,8% SSM
El Euch				30,4%MAL
Tunisie (2010)	46	36	8,34	26%MN
				21,7% SSM
Zouhair			1,5: 2cas	7O% MN
Maroc (2004)	82	25	≥3: 9cas	24%MAL
				4,8%SSM
Notre étude	60	17	10	68,3% MN
				13,3% MAL
				8,3% SSM

La localisation plantaire prédominante dans notre série, et qui l'est dans les séries africaines pouvant aller jusqu’à 80% [10, 6] serait probablement un facteur responsable de cette épaisseur tumorale.

Des études se sont intéressées à rechercher des facteurs associés au développement de mélanomes épais (> 2 et 3 mm), ils ont retrouvé les types nodulaire et acrolentigineux, et les localisations céphalique et des membres inférieurs, en plus de facteurs sociodémographiques qui sont le sexe masculin, l’âge avancé, le bas niveau socioéconomique, les sujets qui vivent seuls et ceux qui n'ont pas été vus par un médecin durant les 3 dernières années avant le diagnostic de mélanome [16, 17].

Des mutations de BRAF, Ckit ou d'autres altérations génétiques peuvent exister expliquant la prédominance de la localisation plantaire, du type nodulaire et l'agressivité de ces mélanomes [18–20, 22]. Tous ces éléments détermineraient un profil génotypique et phénotypique bien distinct.

## Conclusion

Les mélanomes épais sont les plus graves, c'est la forme prédominante dans notre contexte; il nous reste à en définir les facteurs de risque épidémiologiques et génétiques.
